# Pulmonary artery catheter insertion in a case with undiagnosed isolated persistent left superior vena cava

**DOI:** 10.1186/s40981-024-00731-2

**Published:** 2024-07-30

**Authors:** Yoshihiko Chiba, Mineto Kamata, Takuya Ichimura

**Affiliations:** 1 Department of Anesthesiology, Saitama International Medical Center, 1397-1, Yamane, Hidaka-City, Saitama-Pref 350-1298 Japan; 2 Department of Anesthesiology and Critical Care Medicine, Jichi Medical University Saitama Medical Center, Saitama, Japan

To the editor,

The isolated persistent left superior vena cava (PLSVC) is an extremely rare venous malformation characterized by the presence of the PLSVC without the right superior vena cava (SVC). It is found in approximately 0.09–0.13% of patients with congenital heart disease [1] and is typically asymptomatic. Diagnosis is often incidental and can be detected through imaging studies such as computed tomography (CT). We report a case in which transesophageal echocardiography (TEE) was useful in diagnosing such a rare anatomical variation.

A 58-year-old male was transferred to our hospital for emergency surgery due to acute type A aortic dissection. He had been urgently transported to the previous hospital with a chief complaint of sudden-onset chest pain, and a CT scan had confirmed the diagnosis of acute type A aortic dissection. Due to compatibility issues with the image viewing system, the anesthesiologist was unable to review the CT images from the previous hospital. After induction of anesthesia, a TEE probe was inserted, revealing a dilated coronary sinus. The right SVC was not clearly identified, leading to the belief that it was due to compression by the enlarged ascending aorta [2]. Subsequently, as per agreement with the cardiovascular surgery department, a pulmonary artery catheter (PAC) was inserted. A guidewire was first inserted from the right internal jugular vein and confirmed with TEE to be in the right atrium, without noticing that the wire was passing through the coronary sinus. Then, a sheath was inserted, followed by the insertion of a PAC, guided by the pressure waveform. The insertion length of the PAC was 55 cm from the puncture site at the time of completion of placement; therefore, no abnormalities in the route of PAC placement were suspected. After the PAC was inserted without any difficulty, TEE confirmed the placement of the PAC tip in the main pulmonary artery. However, subsequent TEE revealed that the PAC had entered the right atrium through the coronary sinus instead of the SVC (Fig. 1A). Intraoperatively, following pericardiotomy, a defect was identified in the right SVC and diagnosed as an isolated PLSVC, which was overlooked on preoperative CT due to the urgent situation. The PAC revealed no intraoperative issues and was utilized as a route for drug administration and monitoring during the surgery (Fig. 1B). After surgery, the patient was transported to the intensive care unit while intubated. The postoperative course was uneventful, and the PAC was removed on the first postoperative day.

**Fig. 1:**
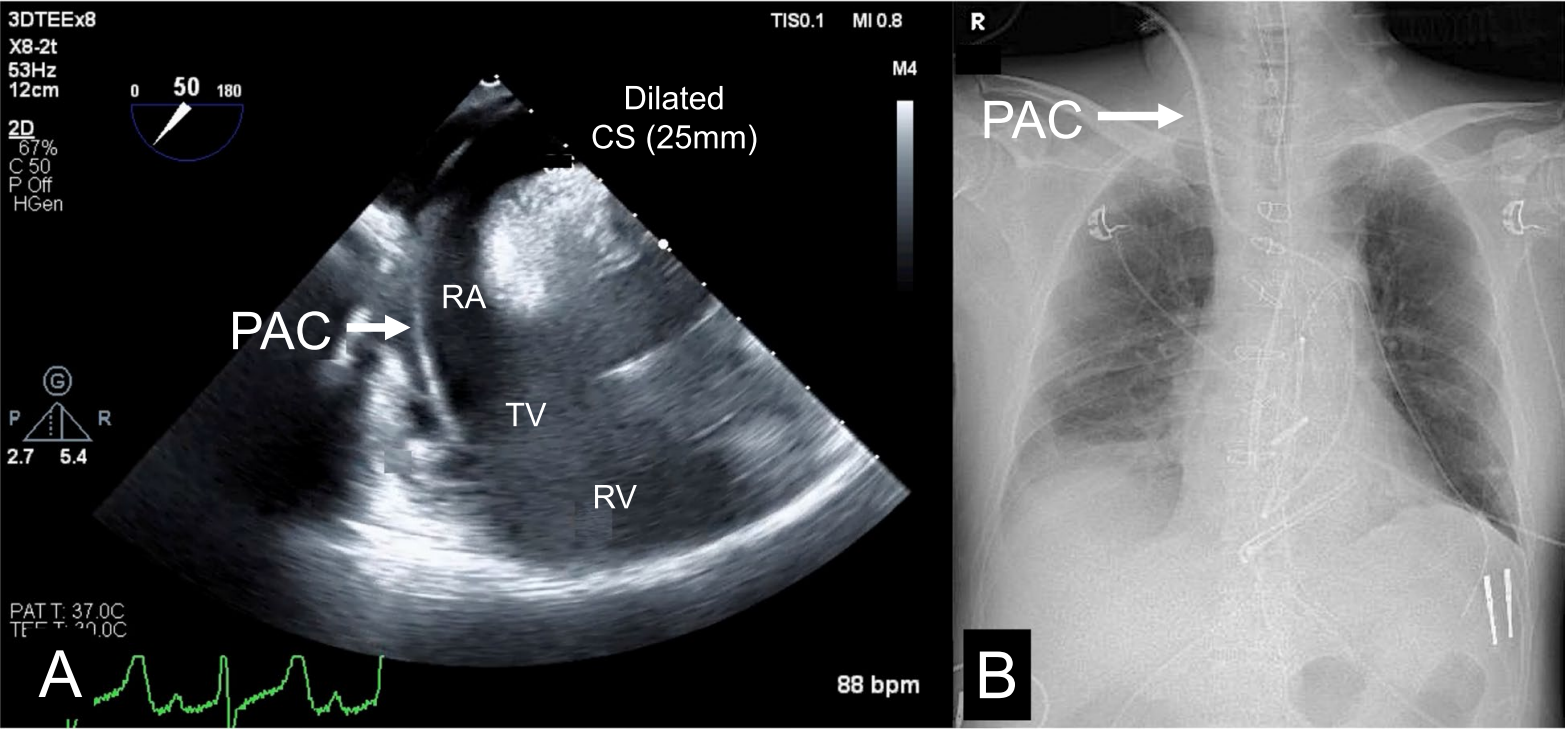
Intraoperative TEE findings and postoperative radiograph. **A** Transesophageal echocardiography image shows the mid-esophageal deep four-chamber coronary sinus view. The pulmonary artery catheter is placed through the coronary sinus and inserted into the right atrium, tricuspid valve, and right ventricle. **B** Postoperative thoracic radiograph taken in the operating room. CS: Coronary sinus. PAC: Pulmonary artery catheter. RA: Right atrium. RV: Right ventricle. TEE: Transesophageal echocardiography. TV: Tricuspid valve

In patients with isolated PLSVC, placement of PAC or other devices via the jugular vein is not only reported to be difficult but also poses the risk of cardiac tamponade due to vascular injury, lethal arrhythmia caused by stretching of the atrioventricular junction resulting from coronary sinus dilation, and acute SVC syndrome [3]. Thus, this is the first reported case of incidental PAC insertion through the internal jugular vein for emergency surgery in a patient with isolated PLSVC.

In this case, the anesthesiologist did not consider the possibility of an isolated PLSVC based on TEE findings prior to PAC insertion. Fortunately, there were no perioperative complications related to the PAC, and it could be safely managed while in place. This case suggests that even without preoperative CT imaging, isolated PLSVC can be diagnosed from TEE findings if one is aware of the possibility.

## Data Availability

Not applicable.

## Abbreviations

CT: Computed tomography
PAC: Pulmonary artery catheter
PLSVC: Persistent left superior vena cava
SVC: Superior vena cava
TEE: Transesophageal echocardiography
